# Visual detection of heart failure associated MiRNA with DSN enzyme-based recycling amplification strategy†

**DOI:** 10.1039/d1ra01500a

**Published:** 2021-05-19

**Authors:** Zhenfei You, Zeping Yang, Yu Chen, Lei Zhang

**Affiliations:** Department of ICU, The Third Affiliated Hospital of Chongqing Medical University (Gener Hospital) Chongqing 401120 China 650265@cqmu.edu.cn zhangleicqmu@163.com

## Abstract

MicroRNA 21 (miRNA-21) is reported to be closely associated with the development of heart failure, cardiac fibrosis and hypertrophy, which could be a promising biomarker for the early-diagnosis of heart failure. Herein, we propose a novel colorimetric miRNA detection method through the integration of duplex-specific nuclease (DSN)-based signal amplification and AuNPs assembly induced colorimetry. Highlights of the proposed method are calculated as: (i) no high temperature hybridization process is required; (ii) DSN enzyme induced recycling provides a favorable detection sensitivity and discrimination of color changes. We believe that the proposed DSN enzyme based colorimetric assay can be applied to heart failure related diagnostics.

## Abstract 2

MicroRNA 21 (miRNA-21) is reported to be closely associated with the development of heart failure, cardiac fibrosis and hypertrophy, which could be a promising biomarker for the early-diagnosis of heart failure.

## Graphical abstract

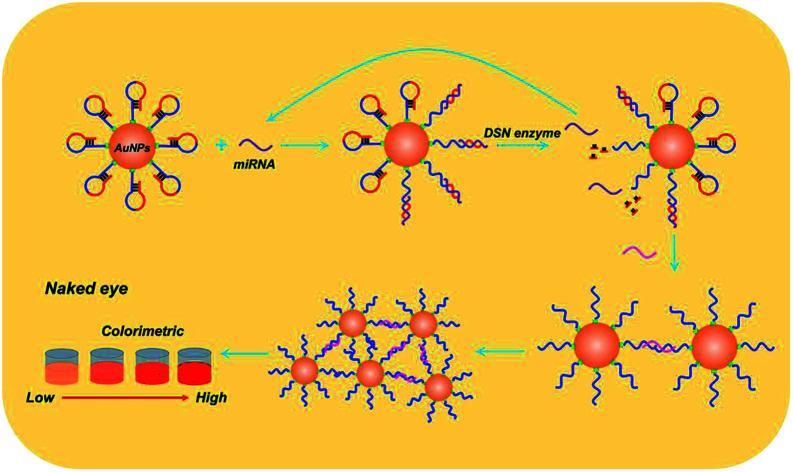

## 1. Introduction

MicroRNAs (miRNAs) are endogenous single-stranded RNAs containing less than 22 nucleotides encoded by short repetitive sequences in the genome.^1–3^ MiRNAs have been intensively reported to be closely associated with a variety of epigenetic processes.^4,5^ MiRNAs play a pivotal role in the formation of heart tissue and are responsible for regulating several phases in cardiac development.^6,7^ Among all the miRNAs discovered, miRNA-21 could enhance tumor growth and also mediate the homeostasis of the cardiovascular system.^8^ The aberrant expression of miRNA-21 is closely related with a number of cardiovascular diseases, including cardiac fibrosis, coronary heart disease and hypertrophy.^9^ Therefore, accurate and sensitive miR-21 detection will provide a valuable means for identifying and evaluating heart failure.

The most conventional miRNA detection methods include northern blotting, quantitative real-time polymerase chain reaction (qRT-PCR) and oligonucleotide microarray.^10,11^ Even though all these method have been demonstrated their feasibility in clinical application, they are also criticized by some shortcomings, such as expensive reagents, time-consuming process, which limited their further application in the field of *in vitro* diagnostics (IVD).^12,13^ In details, microarray was a method developed from northern blotting with advantages of multiplex target detection,^14^ but still bothered by the limited sensitivity and low repeatability between batches. Furthermore, qRT-PCR method could provide a gold standard for miRNA detection due to its high sensitivity and feasibility. However, the qRT-PCR method also suffers from the drawbacks of requirements of specialized equipment and tedious labor-intensive steps. Hence, alternative miRNA detection strategies were in urgently demand to remedy the drawbacks existed in conventional strategies. In recent years, a variety of novel methods have been developed and demonstrated their feasibility for sensitive miRNA detection,^15,16^ including florescence,^17^ colorimetry, electrochemistry^18^ and the next generation sequencing.^19^ For example, duplex-specific nuclease (DSN)-based miRNA detection biosensors have attracted abundant attention due to its special characteristics to specifically degrading dsDNA or DNA–RNA duplexes and inactive toward single-stranded oligonucleotides or dsRNA. The character of DSN enzyme made it a promising option for signal amplification in DSN-based biosensor. However, few researches reported the application of DSN enzyme for colorimetric miRNA detection, which is more likely to be applied for clinical miRNA detection with a more directly result output manner.

Herein, we propose here a novel colorimetric miRNA detection method through the integration of DSN enzyme-based signal amplification and AuNPs assemble induced colorimetry. In the proposed method, the designed AuNPs probe could be recognized target miRNA and thus trigger the signal recycle of target under the assistance of DSN enzymes. Afterwards, the assemble through linker probe could then trigger the color effect of AuNPs. Through directly read the color in the sensing system, we can eventually obtain amounts of miRNA in the detection sample. In all, we believe that the method could provide a new tool for visualized miRNA detection and thus contribute to the diagnosis and prognosis of cardiovascular disease.

## 2. Results and discussions

### 2.1 The working principle of the proposed method for colorimetric miRNA detection

**Scheme 1:**
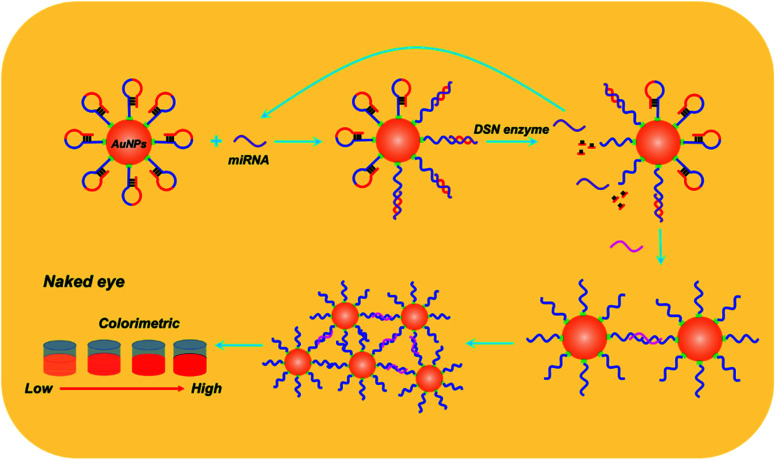
Working principle of the proposed method for colorimetric miRNA detection.

Details of working mechanism of the method is illustrated in Scheme 1. In details, we have designed a stem-loop structure probe and labeled them on the surface of AuNPs. AuNPs based colorimetric detection is a simple and inexpensive form, which is widely used in POCT (Point Of Care Test). The aggregation of AuNPs shifts the absorption peak to a longer wavelength and changes the color of the colloidal solution from red to purple, which can be clearly recognized by the naked eye. When target miRNA existed, it could hybridize with the 3′ terminal (red section) of stem-loop probe and gradually unfold the probe, which consequently form an RNA–DNA double strand. Based on the special characteristics of DSN enzyme to specifically degrading dsDNA or DNA–RNA duplexes and inactive toward single-stranded oligonucleotides or dsRNA. As a result, the DNA sequences section that is complementary with target miRNA is degraded and consequently release miRNA. Released miRNA then participant in the next recycle amplification process to continuously generate RNA–DNA double strand products on the surface of AuNPs. Meanwhile, the recognition section of designed linker is exposed after DSN enzyme-based degradation. Under the assistance of a designed linker, AuNPs aggregated and consequently changes the color of a colloidal solution from red to purple. The color change is positively correlated with the concentrations of amounts of target miRNAs in detection samples.

#### Feasibility of the proposed method for miRNA detection

**Fig. 1:**
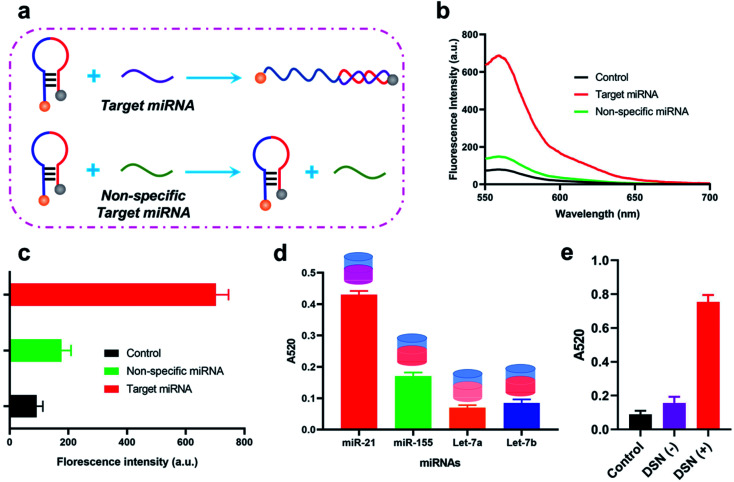
Feasibility of the proposed method for miRNA detection. (a) Illustration of the florescence assay to investigate the interaction between the stem-loop probe and target miRNA. (b) Florescence spectrum of the stem-loop probe when incubated with target miRNA, non-specific miRNA. Control refers the stem-loop probe alone. (c) Calculated florescence intensity of the stem-loop probe when incubated with target miRNA, non-specific miRNA. (d) A52 result of the sensing system for different miRNAs detection. (e) A520 result of the sensing system when DSN enzyme existed or not. Control refers to the florescence probe alone.

We firstly investigated weather designed stem-loop probe could be specially recognized and unfolded by target miRNA through a florescence assay as illustrated in Fig. 1a. In the florescence assay, we have labeled a florescence moiety (Cy3) and corresponding quenching moiety (BHQ-2) on the both terminals of stem-loop probe. Thus, florescence of Cy3 is quenched by BHQ-2 in original stem-loop state of the probe. With the presence of target miRNA, it could hybridize with the 3′ terminal of the probe and gradually unfold it, which consequently lead to the recovery of florescence intensity. From the obtained florescence result in Fig. 1b and c, we have observed a significantly enhanced florescence intensity when stem-loop probe incubated with target miRNA compared with the stem-loop probe alone, suggesting the successful unfold of stem-loop probe by miRNA. With the aims to further investigated the recognition specific of stem-loop probe, we have also applied it for different miRNA identification, including miR-155, Let-7a, Let-7b. Consequently, the obtained florescence intensity when incubated with miR-21 was much higher than the other three miRNAs, indicating the specificity of the designed stem-loop probe for target miRNA-21 identification. In order to study the stability of the stemloop probe, a florescence assay is carried out. In details, the stemloop probe is in its florescence “OFF” state due to the fluorescence resonance transfer between the Cy3 and BHQ-2 that are labeled at the two terminals of stemloop probe. The stability is evaluated through detection of florescence signals when stemloop probe incubated with different regents, including DMEM, PBS buffer and BSA. Consequently, the obtained florescence signals of stemloop probe when incubated with different regents remain the same with that in Control (DEPC water), indicating the probe could stay stable even in complicated experimental conditions (Fig. S1†). We then studied the feasibility of the method for target miRNA detection though calculating the absorbance. As shown in Fig. 1d, when incubated with target miRNA-21, the calculated A520 of whole sensing system was significantly increased compared with the control group. In addition, the calculated A520 of whole sensing system was about 4.5 times higher than that of calculated A520 result when DSN enzyme absent (Fig. 1e). Furthermore, the A520 in the absence of DSN enzyme is 1.5 fold higher than the control group which could be explained by that there were possible some aggregation of AuNPs after the unfold of stemloop probe by target miRNA. In the method, DSN enzyme play an important role in the recycling amplification and also responsible for the following aggregation of AuNPs.

#### Optimization of the experimental conditions

**Fig. 2:**
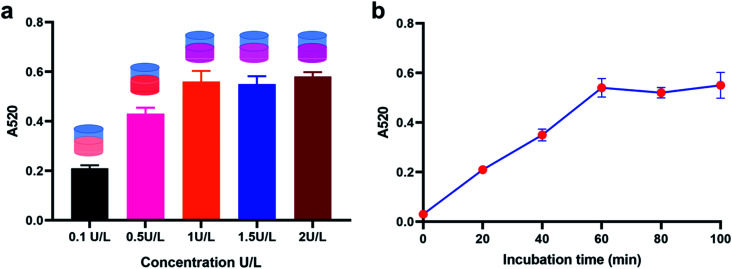
Experimental conditions optimization of the proposed method for miRNA detection. (a) Calculated A52° result of the method with different concentrations of DSN enzymes. (b) Calculated A52° result of the method with incubation time.

We then optimized the experimental conditions of the proposed method for a better sensing performance. First of all, we investigated the concentration of DSN enzyme utilized in the sensing system through observing the calculated A520 result for 100 nM miRNA-21 detection when incubated with different concentrations of DSN enzymes from 0.1 U L^−1^ to 2 U L^−1^. Consequently, the obtained A520 result gradually increased with the increase of DSN enzymes concentrations ranging from 0.1 U L^−1^ to 1 U L^−1^, and no more increases were observed when the concentration more than 1 U L^−1^, demonstrating that 1 U L^−1^ of DSN enzyme could provide a favorable detection performance (Fig. 2a). Afterwards, we optimized the incubation time of DSN enzyme. From the result in Fig. 2b, the calculated A520 result is positively correlated with the incubation time before 1 hour and no attached increases were observed when incubated with more than 1 hour, suggesting that incubated with DSN enzyme for about 1 hour could provide an optimized detection result.

#### Sensitivity and specificity of the proposed method for miRNA detection

**Fig. 3:**
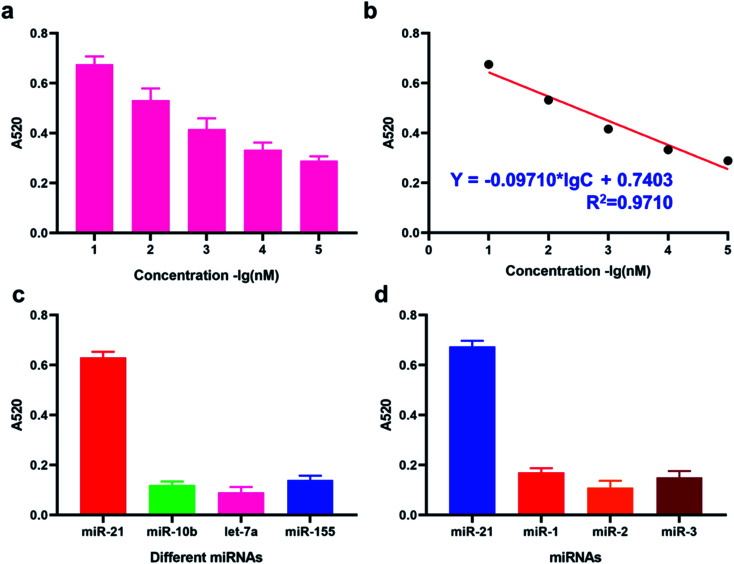
Sensitivity and specificity of the proposed method for miRNA detection. (a) Calculated A520 result of the method with different concentrations of miRNAs. (b) Correlation equation of the calculated A520 result with miRNA of different concentrations. (c) Calculated A520 result of the method for different miRNAs detection. (d) Calculated A520 result of the method for different miRNAs detection.

Under the optimized experimental condition, we have then investigated the sensitivity of the DSN enzyme-based colorimetric method though applying it for the detection of target miRNA-21 with different concentrations ranging from 1 pM to 1 nM. From the result in Fig. 3a, the calculated A520 result is positively correlated with the concentrations of target miRNA-21. The obtained equation between calculated A520 result and amounts of target miRNA-21 was *Y* = −0.09710 × lg *C* + 0.7403 with the correlation coefficient 0.9710. LOD (Limit Of Detection) of the method for miRNA-21 detection was determined 29 fM level through 3*σb*/slope method, which is comparable or even superior to that of the former proposed colorimetric methods (Table 1). Moreover, the proposed method also exhibited a favorable selectivity towards target miRNA-21 (1 nM) from a mixture of interferences at the same concentration (miRNA-155, miRNA-10b and let-7a) (Fig. 3c). Despite that a good specificity of the method was obtained, analysis of target miRNA from miRNAs with highly related sequences remains a huge challenge. We thus applied the method for the distinguishment of highly similar miRNAs (miRNA-1, miRNA-2, and miRNA-3) with single or double base mutation (Fig. 3d). The obtained florescence result indicated that the established method enables miRNA detection with single-base resolution, which would particularly contribute to the analysis of a miRNA family with a single-base sequence difference.

**Table 1:** A brief comparison between the proposed method with former miRNA detection strategies^a^

Title	Signal type	Sensitivity	Signal amplification	Ref.
The method	Colorimetry	29 fM	DSN enzyme based recycle	
DNAzyme-based	Colorimetry	1 pM	HCR	20
CRISPR-Cas based	Florescence	fM	RCA	21

a HCR, hybridization chain reaction; RCA, rolling circle amplification.

#### Application of DSN enzyme-based colorimetric method for exosomal miRNA-21 detection

**Fig. 4:**
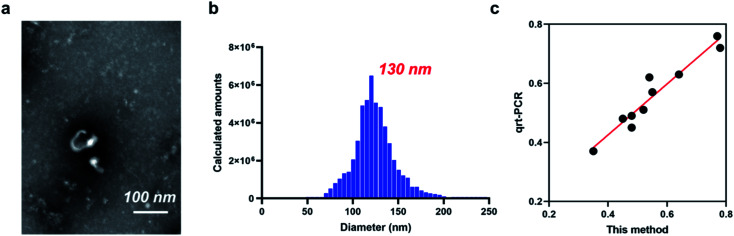
Application of DSN enzyme-based colorimetric method for exosomal miRNA-21 detection. (a) TEM result of the extracted exosomes. (b) NTA of the extracted exosomes. (c) Correlation relation of the proposed method and qRT-PCR.

To investigate the potential of DSN enzyme-based colorimetric method for exosomal miRNA-21 detection, we quantified miRNA-21 in exosomes isolated from the clinical serum sample of patients with heart failure. Transmission electron microscope (TEM) and nanoparticle tracking analysis (NTA) were utilized for morphological observation of the isolated exosomes. The obtained TEM result indicate that the extracted exosomes exhibited a flatted round shape (Fig. 4a). The diameters of the isolated exosomes varied from 78 nm to 210 nm (Fig. 4b) and the peak value was 115 nm, which is consistent with the previous reports. To verify the detection performance of the method, the extracted exosomal let-7a is diluted into different concentration and detected by both the method and RT-qPCR. As expected, the exosomal miRNA-21 detection results through the proposed approach maintained a high consistency with the results from RT-qPCR (*R*^2^ = 0.946) (Fig. 4c), suggesting that the proposed method has a high application potential in detecting clinical specimens.

## 3. Conclusion

AuNPs based colorimetric miRNA detection methods have been developed for more than 20 years. However, the colorimetric methods are suffer from the limitations of low discrimination of color changes, requirements of high hybridization temperature. We proposed here a novel colorimetric miRNA detection method with DSN enzymes for signal recycles. Based on this, the proposed method has achieved several critical advances: (a) free from high temperature hybridization process; (b) DSN enzyme induced recycle provide a favorable detection sensitivity and discrimination of color changes. We believe that the proposed DSN enzyme based colorimetric assay can be applied to heart failure related diagnostics.

## Conflicts of interest

There are no conflicts to declare.

## Supplementary Material

RA-011-D1RA01500A-s001
