# Supplementary material for: Visual detection of heart failure associated MiRNA with DSN enzyme-based recycling amplification strategy
Source: RSC Adv. 2021 May 19;11(29):18068–73. doi: 10.1039/d1ra01500a (PMC9033192; doi:10.1039/d1ra01500a)
Supplement: RA-011-D1RA01500A-s001 [file RA-011-D1RA01500A-s001.pdf]

Electronic Supplementary Material (ESI) for RSC Advances.
This journal is © The Royal Society of Chemistry 2021

Supporting Information

Visual Detection Of Heart Failure Associated MiRNA With DSN Enzyme-Based Recycling Amplification Strategy

Zhenfei You[1], Zeping Yang[1], Yu Chen, Lei Zhang*

Author affiliation:
Department Of ICU, The Third Affiliated Hospital of Chongqing Medical University (Gener Hospital), Chongqing, 401120, China.

***Corresponding author:**
Dr Lei Zhang, Department Of ICU, The Third Affiliated Hospital of Chongqing Medical University (Gener Hospital), Chongqing, 401120, China.
E-mail: 650265@cqmu.edu.cn;

Experimental Methods

Materials and Apparatus: All chemicals were supplied by Chongqing Chemical Reagent Factory (Chongqing, China). The enzymes and dNTPs used for RNA transcription and the buffers were purchased from Bio-Lifesci (Guangzhou, China). PCR reagents were purchased from Takara (Tokyo, Japan). the fluorescent double-labeled probe was synthesized by Takara (Japan). All DNA and RNA sequences used in this study are provided in **Table S1**. $HAuCl_4$ solution was obtained from Aladdin (Shanghai, China). Reagents used for protein expression and purification were purchased from Abiotech (Jinan, China). Fluorescence detection was performed on a thermal cycler Dice™ Real Time System III (Takara, Japan). Low-speed centrifugation experiment was carried out using a mini benchtop centrifuge (Sangon Biotech, Shanghai, China). High speed centrifugation and PCR experiments were performed using the instruments from Eppendorf. Polyacrylamide gel electrophoresis (PAGE) experiments were performed using a Beijing Liuyi instrument and imaged by a gel imager (Baijing, Beijing, China). All other buffers and solutions were prepared using ultrapure water (>18.25 MΩ).

Synthesis and Characterization of AuNPs: AuNPs were prepared by the sodium citrate reduction method as reported previously. Before the experiment, the glassware was immersed in aqua regia (HNO3:HCl = 1:3) for over 30 minutes and then rinsed with ultrapure water. 100 mL of 1 mM HAuCl4 was added into a 250 mL flask and heated to boiling on a heated magnetic stirrer. After boiling became stable, 10 mL of 38.8 mM sodium citrate solution was added with rapid stirring. The solution became colorless after a short time. After 20 minutes, the solution turned wine red and the heating was stopped. Then, the solution was cooled down to room temperature to obtain AuNPs solution (13 nm in diameter).

Labeling of AuNPs-DNA Probes by Freezing: 50 μL of 100 μM poly A-tagged DNA probes was added to 1 mL of AuNPs solution. The solution was mixed and frozen in a refrigerator (–20°C). The freezing time was at least 2 hours. After thawing, the solution was centrifuged at 4°C at 12,000 rpm for 30 minutes. The supernatant was aspirated and the precipitate was resuspended in a wash buffer (0.1 M NaCl in 0.01 M phosphate buffer, pH 7.4). This step was repeated three times. Finally, the pellet was S5resuspended in a buffer (0.3 M NaCl in 0.01 M phosphate buffer, pH 7.4) and the AuNPs-DNA probes were stored at 4°C in the dark.

Labeling of AuNPs-DNA Probes by Freezing: 50 μL of the obtained AuNPs probe complex was mixed with target miRNA with different concentrations. After incubation for about 30 min, we have then added 5 μL linker to the mixture and incubated for another 60 min.

miRNA detection with DSN enzyme-based sensor: 50 μL of the obtained AuNPs probe was then mixed with different concentration of target miRNA for 30 minutes at room temperature. Afterwards, 5 μL purchased DSN enzymes were then added in the mixture and incubated for 1 hour at 37°C. 5 μL linker probes (10 μM) were then added the in the mixture and incubated for another 30 minutes and the color change is quantified by the Loverbourne colorimeter (ST110).

Exosome extraction and characterization: HCM cells were cultured in RPMI-1640 medium with 10% fetal bovine serum (FBS) and maintained in a humidified atmosphere of 5% CO_2 at 37°C. For EV isolation, we washed the cells three times with phosphate-buffered saline (PBS) when cells reached 70% confluence and maintained them for an additional 12 hours in medium without FBS; the culture medium was then collected for EV isolation by ultracentrifugation according to standard differential centrifugation separation protocols. Finally, transmission electron microscopy (TEM), nanoparticle tracking analysis and flow cytometry analysis were

performed to characterize these isolated EVs.

Statistical Analysis: Each test was repeated at least three independent replicates, which were displayed as the mean ± standard deviation (SD). Data were visualized using software GraphPad Prism 8.0 (CA, USA). The Student's t test was used to analyze comparisons between two groups. Multiple groups were compared with one-way ANOVA and Least-Significant Difference method (LDS). Differences were considered significant at values of $P < 0.05$.

Table S1. the details of nucleic acids sequences used in the sensing system

Title	Sequence (5'-3')
Probe-1	AAAAAAAAAATTTTTATGATGTTCGTTGTGAuNPs-DNA
Probe-2	AAAAAAAAAATTTTTCGTTTAGGATTTGT
miRNA-21	UAGCUUAUCAGACUGAUGUUGA
miRNA-155	UUAAUGCUAAUCGUGAUAGGGGU
miRNA-10b	UACCCUGUAGAACCGAAUUUGUG
miRNA-1	UAGCUUAGGUGACUGAUGUUGA
miRNA-3	UAGCUUACAAGACUGCUGUUGA
miRNA-3	UAGCUUACCAGACUUCUGUUGA

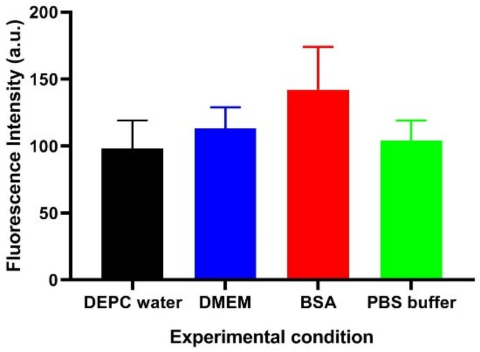

Figure S1. Florescence intensity of the stemloop probe when incubated with different regents.
